# Reducing Rates of Cesarean Delivery in Rural US Communities: A Systematic Review of Interventions and Approaches to Care

**DOI:** 10.5888/pcd23.260039

**Published:** 2026-07-30

**Authors:** Jena L. Funakoshi, Hannah M. Collins-Doijode, Kathryn L. Braun

**Affiliations:** 1 Department of Public Health Sciences, University of Hawaiʻi at Mānoa, Honolulu, Hawaiʻi; 2 John A. Burns School of Medicine, University of Hawaiʻi at Mānoa, Honolulu, Hawaiʻi

## Abstract

**Introduction:**

Cesarean deliveries are the most common major surgery in the US, with rates rising disproportionately in rural communities. While sometimes medically necessary, unnecessary cesarean births increase risks for maternal death, long-term complications, and intergenerational health effects that contribute to the burden of chronic disease. This systematic review examined studies describing interventions and approaches to care that reported outcomes related to reducing cesarean delivery rates in rural US communities.

**Methods:**

We searched 4 databases in September 2025. Studies were eligible if they were conducted in rural US settings and reported cesarean-related outcomes associated with an intervention or care approach. We categorized interventions as patient level, provider level, or health system level. Two reviewers independently screened articles for inclusion, extracted data, and assessed study quality using the Newcastle-Ottawa Scale and the Joanna Briggs Institute checklist.

**Results:**

Nine studies met inclusion criteria. Of the 2 patient-level interventions, psychosocial education was associated with lower cesarean delivery rates (21% in intervention vs 40% in control), whereas a mobile health application showed only a marginal difference (27.1% among application users vs 27.7% among nonusers). None were categorized at the provider level. Seven interventions tested system-level models, primarily comparing hospitals with different staffing patterns; family medicine–led hospitals had lower rates of low-risk nulliparous, term, singleton, vertex cesarean delivery than hospitals staffed by both family medicine physicians and obstetricians (23% vs 28%), certified nurse-midwife–managed births had lower cesarean delivery rates than family medicine physician–managed births (8% vs 14%), and collaborative maternity care models integrating midwives, nurses, and obstetricians were associated with cesarean delivery rates declining from 26.2% to 11.2%.

**Conclusion:**

System-level approaches, particularly those that restructure maternity care teams, emphasize family medicine physician–led models, and integrate midwifery and culturally grounded childbirth practices, are more consistently associated with lower cesarean delivery rates in rural US settings than patient-level interventions alone. Future efforts to reduce unnecessary cesarean deliveries should prioritize strategies tailored to the variability of rural care capacity.

**Summary**

**What is already known on this topic?**

Rural US communities have higher rates of cesarean delivery compared with urban areas because of limited access to maternity care and shortages of physicians, midwives, and anesthesia services.

**What is added by this report?**

In this systematic review of 9 studies, patient-level interventions had varying associations with rates of cesarean delivery, whereas health system–level approaches, particularly those involving staffing models and care team structure, had more consistent associations with lower rates of cesarean delivery.

**What are the implications for public health practice?**

Reducing the rate of unnecessary cesarean deliveries in rural communities requires aligning interventions with local resource capacity and health system context.

## Introduction

Cesarean delivery is the most common major surgery in the US, accounting for nearly 1 in 3 births each year (1). While cesarean delivery can be lifesaving, non–medically indicated cesarean deliveries expose infants to unnecessary risks, including the development of chronic immune conditions, asthma, obesity, and other noncommunicable diseases (2). These risks may occur in part because infants born via cesarean delivery are less exposed than infants born vaginally to maternal vaginal microbiota, which may affect the establishment of the neonatal microbiome and immune system development (2). For mothers, cesarean deliveries increase the risk of poor wound healing, hemorrhage, infection, and complications in future pregnancies (3).

Despite these risks, cesarean delivery rates continue to rise. Globally, 21.1% of women give birth by cesarean delivery (4). In the US, Healthy People 2030 set a goal to reduce cesarean deliveries among low-risk nulliparous, term, singleton, vertex (NTSV) births from 25.9% in 2018 to 23.6%, yet data from 2022 showed that rates instead increased to 26.3% (5). National trends, however, obscure disparities between urban and rural health care systems. Each year, approximately half a million women give birth in rural US hospitals, yet more than half of rural communities lack hospital-based birthing services (6,7). During the past decade, more than 100 rural hospitals closed, and many discontinued birth services (8). Long travel distances for maternity care are associated with worse outcomes, more medical interventions, and a higher risk of preterm birth (8).

Although previous systematic reviews identified patient-, provider-, and system-level strategies that may help reduce potentially avoidable cesarean deliveries, most evidence comes from hospitals in urban or mixed settings. Rural settings, which face distinct workforce, access, and infrastructure challenges, are understudied. This study aimed to systematically review studies describing interventions, care approaches, and reporting outcomes related to reducing cesarean delivery rates in rural US communities. Because few studies distinguished between medically indicated and non–medically indicated cesarean deliveries, overall cesarean delivery rate was used as the primary outcome and interpreted cautiously as a proxy for potentially unnecessary cesarean delivery when indication was not specified.

## Methods

We followed the Preferred Reporting Items for Systematic Reviews and Meta-Analyses guidelines in reporting the results of this study (9). This review was not prospectively registered because it was initially conducted as part of an academic assignment. However, we defined systematic review methods in advance, including the research question, eligibility criteria, search strategy, and data extraction procedures, to ensure methodologic rigor and transparency.

### Data sources and search terms

We conducted a systematic search of PubMed, CINAHL, Cochrane Library, and Web of Science Core Collection in September 2025 (Appendix). The search strategy used predefined terms related to interventions, care approaches, cesarean section, rural populations, and the US. Search terms were adapted for each database using the Polyglot Search Translator (10).

### Study selection

Studies were included if they 1) were conducted in the US, 2) examined an intervention or care approach in a rural setting, and 3) reported outcomes related to reducing cesarean delivery rates. Studies were eligible if they focused on patient-level (defined as patient education or counseling), provider-level (provider-targeted training and/or practice recommendations), or health system–level strategies (staffing patterns, care models, and hospital policies). We applied no date limits and excluded studies if they focused exclusively on nonrural populations, did not report cesarean-related outcomes, or were conducted outside the US. Only English-language publications were considered.

Definitions of “rural” vary across datasets and classification systems. This review did not apply a single standardized definition of rural (eg, Rural–Urban Commuting Area codes or US Census–based classifications). Instead, we included studies if the authors explicitly identified the study population, setting, or participating sites as rural. For studies conducted in mixed or unspecified settings, we sought additional clarification from authors.

After identifying eligible studies, we removed duplicate studies and then used Rayyan (11) to track article exclusion at the title, abstract, and full-text review stages.

### Quality appraisal

We assessed methodologic quality using the 9-star Newcastle–Ottawa Scale for the retrospective cohort design studies (12) and the 13-item Joanna Briggs Institute checklist for randomized control trials (13). For the Newcastle–Ottawa Scale, we classified studies as good quality if they received 3 or 4 stars in the Selection domain, 1 or 2 stars on the Comparability domain, and 2 or 3 stars in the Outcome domain; fair quality if they received 2 stars in Selection, 1 or 2 stars in Comparability, and 2 or 3 stars in Outcome; and poor quality if they received 0 or 1 star in Selection, 0 stars in Comparability, or 0 or 1 star in Outcome. For the Joanna Briggs Institute checklist, we assessed each item as yes, no, “unclear,” or not applicable across 13 items. Responses of yes were assigned 1 point, while no and “unclear” responses were assigned 0 points, for a possible score of 0 to 13. Studies were categorized as high quality if they scored 10 to 13, moderate quality if they scored 7 to 9, and low quality if they scored 0 to 6. Two reviewers (J.L.F., H.M.C-D) independently evaluated each study and resolved discrepancies through discussion.

## Results

We identified 507 records through database searches. After removing duplicates, we screened the titles and abstracts of 435 articles and excluded 420 articles. The remaining 15 articles were read in full. We found an additional study through citation chasing. Of these 16 studies, we excluded 7 (2 for wrong outcome and 5 for wrong population), yielding 9 studies (Figure).

**Figure:**
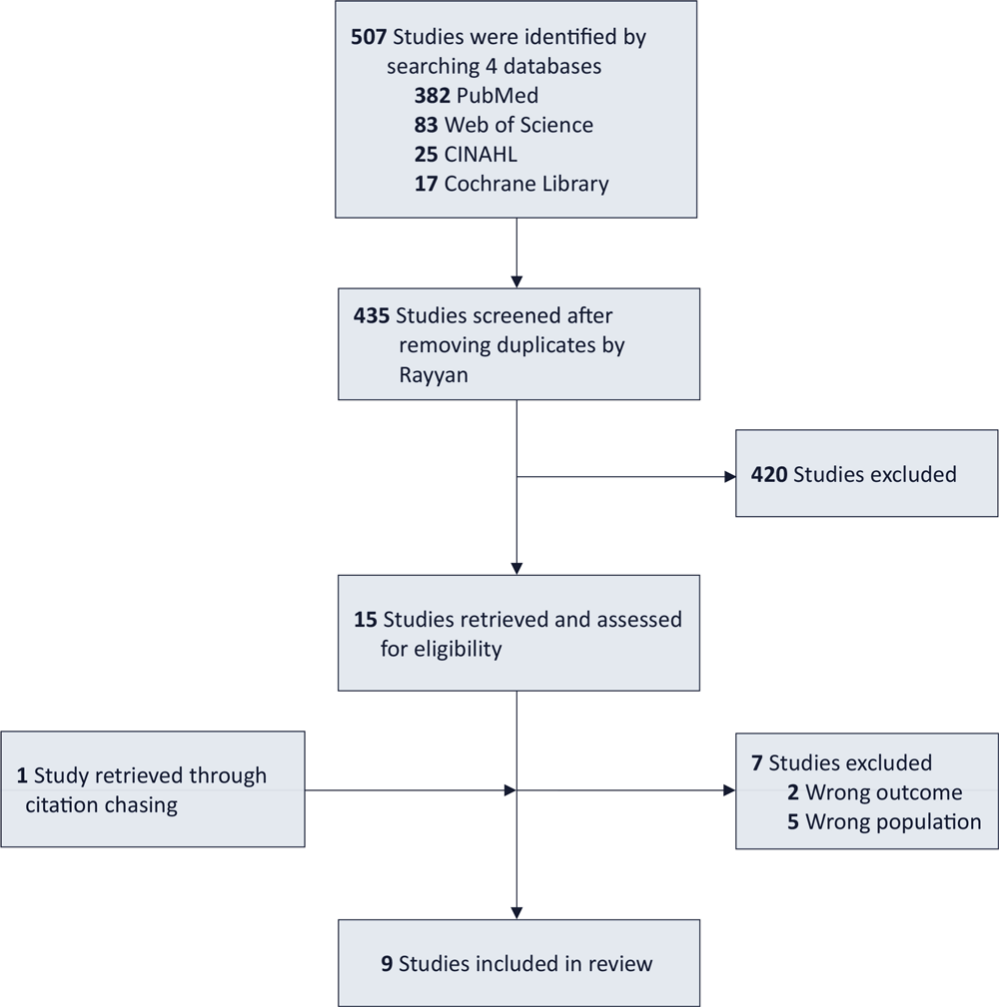
Preferred Reporting Items for Systematic Reviews and Meta-Analyses flowchart for systematic review of studies describing interventions for reducing rates of cesarean delivery in rural US communities.

### Study and intervention characteristics

Seven studies were from single states: Iowa (14), Kentucky (15), New Mexico (16), New York (17), North Carolina (18), Pennsylvania (19), and Wyoming (20) (Table 1). Two studies examined rural birthing units across multiple US states (6,21). Three studies were conducted in both rural and urban areas (14,19,21).

**Table 1:** Characteristics of Included Studies in Systematic Review of Studies Describing Interventions for Reducing Rates of Cesarean Delivery in Rural US Communities

Citation	Location	Population and sample size	Key features
**Patient-level interventions **			
Bush et al (20), 2017	Wyoming	Medicaid-enrolled pregnant women using prenatal mobile app; 85 app users vs 5,158 nonusers	• Compared Medicaid-enrolled pregnant women who used a prenatal mobile health app with Medicaid-enrolled pregnant women who did not use the app. • App designed to support prenatal engagement through pregnancy education, Medicaid benefit information, health milestone content, and app-based resources. • Outcomes measured included 6-month prenatal visit completion, cesarean delivery, low birth weight, and neonatal intensive care unit admission following delivery.
Feinberg et al (19), 2015	Pennsylvania	Expectant, first-time parent couples; 76 couples enrolled in group sessions vs 71 couples not enrolled	• A couples-based transition-to-parenthood program included prenatal and postpartum group sessions focused on coparenting, emotional support, relationship communication, and preparation for parenthood. • Outcomes measured included delivery mode, birth weight, gestational age, pregnancy complications, and maternal and infant hospital length of stay.
**Systems-level interventions**			
Carlough et al (18), 2021	North Carolina	18 rural hospitals with maternity units	• Examined staffing and operational structures in rural maternity units. • Used hospital surveys, site visits, and secondary state-level data to describe health care provider mix, anesthesia coverage, nursing models, surgical capacity, risk and transfer protocols, and maternity unit operations.
Hueston and Rudy (15), 1993	Kentucky	Patients whose labor or delivery was managed by certified nurse–midwives or family medicine physicians	• Compared labor and delivery management among patients whose care was managed by certified nurse-midwives or family medicine physicians in a rural Kentucky co-practice. • Examined delivery route, labor management practices, episiotomy, lacerations, analgesia, and neonatal outcomes by managing provider type.
Prasad et al (6), 2018	9 US states	244 Rural hospitals and their maternity health care providers	• Linked a rural obstetric workforce survey from 9 states with all-payer hospital discharge data. • Examined whether hospital employment of maternity care physicians and physician specialty mix were associated with NTSV cesarean delivery, non–medically indicated cesarean delivery, and non–medically indicated labor induction.
VanGompel et al (14), 2024	Iowa	849 maternity health care providers across 39 hospitals	• Surveyed physicians, nurses, and midwives working in Iowa hospital intrapartum units and linked survey responses with hospital characteristics and outcomes data. • Hospitals compared by whether labor and delivery units included family medicine physicians only, obstetricians only, or both family medicine physicians and obstetricians. • Also examined labor and delivery unit culture and safety culture.
Leeman and Leeman (16), 2003	Zuni Pueblo and Ramah Navajo communities, New Mexico	Predominantly Native American rural birth cohort (n = 1,132). Historical control.	• Examined births in Zuni Pueblo and Ramah Navajo communities from 1992 through 1996. • Studied delivery type, indications for cesarean delivery, obstetric risk factors, labor induction and augmentation, perinatal system design, and cultural factors related to childbirth.
Jolles et al (21), 2020	Birth centers across the US	88,574 Birth center clients (20,371 rural) served by 82 centers (28 rural). Historical control.	• Used the American Association of Birth Centers Perinatal Data Registry to examine the birth center model of care in rural and urban settings. • Compared maternal and neonatal outcomes among clients receiving care in birth centers by geographic setting while accounting for sociodemographic and medical risk factors.
Stone et al (17), 1996	Rural Upstate New York	Pregnant women receiving care in a hospital-based certified nurse–midwife-led service. Historical control.	• Described implementation of a rural hospital–based certified nurse-midwife service within a collaborative maternity care model. • Model integrated certified nurse-midwives, nurses, and obstetricians and emphasized coordinated care, patient education, continuity, and collaborative management.

Abbreviations: app, application; NTSV, nulliparous, term, singleton, vertex.

Of the 9 studies, none reported on a provider-level approach, 2 studies reported on a patient-level approach, and 7 studies on a systems-level approach. At the patient level, Bush et al used a retrospective cohort design to compare outcomes of Medicaid enrollees in Wyoming, a predominantly rural state, who used a mobile health application (app) with those who did not (20). The app was designed to enhance prenatal engagement through appointment reminders, pregnancy education, and symptom tracking. Feinberg et al conducted a randomized controlled trial in rural Pennsylvania that tested a couples-based psychosocial support program involving 9 prenatal and postpartum group sessions fostering emotional support, relationship communication, and coparenting skills for first-time expectant parents (19). In these studies, samples were small, with 85 app users (vs 5,158 nonusers) and 76 couples enrolled in the group sessions (vs 71 not enrolled).

Of the 7 system-level studies, Stone et al used a time-series design to evaluate changes in cesarean delivery rates following implementation of a collaborative maternity care model in a rural hospital in upstate New York (17). The model integrated nurse–midwives, nurses, and obstetricians to provide coordinated, team-based care emphasizing patient education, shared decision-making, and continuity of care.

The other 6 studies used retrospective cohort designs. Prasad et al used an all-payer claims database from 9 US states to examine how employment status and provider specialty mix were associated with NTSV cesarean delivery and non–medically indicated cesarean delivery rates (6). Hueston and Rudy compared outcomes among patients receiving care from family medicine physicians versus certified nurse–midwives in a rural Kentucky practice (15). Jolles et al assessed outcomes from midwifery-led birth centers across 28 rural US sites (21). VanGompel et al examined state-level vital records from 39 rural Iowa hospitals, including a subgroup analysis of 29 hospitals with fewer than 1,000 annual deliveries, by health care provider category (14). Similarly, Carlough et al examined structural and staffing patterns of rural maternity units in North Carolina hospitals (18). Leeman and Leeman examined all births from 1992 through 1996 in the predominantly Native American Zuni Pueblo and Ramah Navajo communities of New Mexico, which maintained a very low cesarean delivery rate of 7.3% (16).

### Intervention outcomes

All 9 studies reported cesarean delivery rates, and 7 studies also reported other outcomes (Table 2). The intervention described by Feinberg et al demonstrated the largest absolute between-group difference in cesarean delivery rates, with cesarean delivery occurring in 40% (29 of 72) of the control group compared with 21% (15 of 71) of the intervention group, an absolute difference of 19 percentage points (19). In addition, those who participated in the group sessions realized improved postpartum mental health, reduced maternal stress, and enhanced coparenting quality. The study describing the mobile health app in Wyoming found that users had a marginally lower cesarean delivery rate compared with nonusers (27.1% vs 27.7%); however, app use was associated with higher rates of completion of the 6-month prenatal visit and a reduced incidence of low birth weight (20).

**Table 2:** Intervention Outcomes in Systematic Review of Studies Describing Interventions for Reducing Rates of Cesarean Delivery in Rural US Communities

Study	Outcome in cesarean delivery rate	Effect estimate, %^a^	Other maternal outcomes
Bush et al (20), 2017	App users had a similar cesarean delivery rate compared with nonusers: 27.1% among app users vs 27.7% among nonusers.	2.2^b^	App use was associated with higher completion rate of a prenatal visit at 6-months or later. Rate of low birth weight was lower among app users.
Feinberg et al (19), 2015	Cesarean delivery occurred in 21% of participants in the intervention group compared with 40% of participants in the control group.	47.5	Intervention was associated with attenuation of the negative association between maternal cortisol on birth weight, gestational age, and days in hospital for infants (adverse birth outcomes).
Carlough et al (18), 2021	Hospitals staffed only by family medicine physicians had a cesarean delivery rate of 24%, hospitals with high proportions of certified nurse–midwives had a cesarean delivery rate of 22%, and hospitals staffed only by obstetricians had a cesarean delivery rate of 32%.	25	Critical access hospitals were more likely than larger rural hospitals to accept patients choosing trial of labor after cesarean delivery (ie, a planned attempt to deliver vaginally after a previous cesarean section).
Hueston and Rudy (15), 1993	Among primiparous women, cesarean delivery occurred in 8% of those managed by certified nurse–midwives compared with 14% of those managed by family medicine physicians.	43	Care administered by certified nurse–midwives was associated with fewer episiotomies compared with care administered by family medicine physicians.
Prasad et al (6), 2018	Rural hospitals that employed family medicine physicians directly (rather than contracting with them) were associated with a 3.9% lower rate of NTSV cesarean deliveries and a 4.0% lower rate in non–medically indicated cesarean deliveries.	NA^c^	Hospital employment of maternity care physicians was not significantly associated with non–medically indicated induction in family physician–only hospitals.
VanGompel et al (14), 2024	Family medicine–only hospitals had a 34.3% lower adjusted risk of NTSV cesarean delivery compared with hospitals staffed by both family medicine physicians and obstetricians. Across all hospitals, mean NTSV cesarean delivery rates were 23% in family medicine-only hospitals, 28% in hospitals with both family medicine physicians and obstetricians, and 23% in obstetrician-only hospitals.	34.3	Nurses at family medicine–only hospitals reported unit norms more supportive of vaginal birth and stronger safety culture compared with nurses at hospitals with both provider types or obstetrician-only staffing.
Leeman and Leeman (16), 2003	The Zuni-Ramah population had a total cesarean delivery rate of 7.3% compared with the 1996 national rate of 20.7%. The primary cesarean delivery rate was 5.3% compared with the 1996 national rate of 14.6%.	65	Trial of labor after cesarean delivery was attempted in 93% of pregnancies among women, compared with 42% nationally. The study population also had lower rates of induction, oxytocin augmentation, and operative vaginal delivery than national rates.
Jolles et al (21), 2020	Rural birth center clients had a cesarean delivery rate of 8.1% compared with 9.6% among urban birth center clients. Adjusted analyses showed no significant difference in odds of cesarean delivery between rural and urban settings.	15.6	Rural birth center clients had low rates of episiotomy and induction and high rates of exclusive breastfeeding at discharge.
Stone et al (17), 1996	Cesarean delivery rates declined from 26.2% before implementation of the certified nurse-midwife service to 11.2% after implementation.	57.3	The new care model was associated with a higher number of women served each year, higher number of twin gestations birthed vaginally, lower number of episiotomies, and lower number of third- and fourth-degree lacerations.

Abbreviation: NA, not applicable; NTSV, nulliparous, term, singleton, vertex.

a Unless otherwise indicated, all values were reported as significant.

b Not significant.

c Prasad et al (6) reported adjusted marginal effects rather than a relative risk reduction.

Among the health system-level approaches, Prasad et al found that in a cross-sectional analysis of rural hospitals, those that directly employed family physicians rather than contracted with them had 3.9 percentage-point lower NTSV cesarean rates and 4.0 percentage-point lower non–medically indicated cesarean rates (6). This employment model was associated with higher physician retention and improved access to obstetric care in rural settings. Furthermore, in hospitals with both family medicine physicians and obstetricians, employing a 10% higher proportion of obstetricians was associated with a 4.6% higher rate of NTSV cesarean delivery (6). Similarly, Van Gompel et al found that the mean NTSV cesarean delivery rate was 23% at hospitals staffed only by family medicine physicians compared with 28% at hospitals staffed by both family medicine physicians and obstetricians, corresponding to a 34.3% lower adjusted risk of cesarean delivery at family medicine–only hospitals (14). Hueston and Rudy found that primiparous women managed by certified nurse–midwives had lower cesarean delivery rates (8%) than those managed by family medicine physicians (14%), and that certified nurse–midwives also used fewer interventions such as analgesia or episiotomy (15). Carlough et al found that hospitals staffed only by family medicine physicians had a cesarean delivery rate of 24%, while hospitals with high proportions of certified nurse-midwives had the lowest rate, at 22%, compared with 32% in hospitals staffed only by obstetricians. Also, using certified registered nurse anesthetist–based anesthesia, cross-trained nursing staff, and flexible staffing models supported safety and effectiveness in rural settings (18).

Stone et al reported that cesarean delivery rates fell from 26.2% (1966) to 11.2% (1994) in rural New York after the hospital adopted a collaborative maternity care model integrating midwives, nurses, and obstetricians (17). Additionally, this program was associated with increased success in vaginal birth after cesarean delivery, fewer episiotomies, and overall improved maternity outcomes (17). Jolles et al found that midwife-led birth centers in rural settings had a cesarean delivery rate of 8.1%, compared with 9.6% in urban birth centers, with adjusted analyses showing no significant difference in odds of cesarean delivery between rural and urban settings (21). Both studies were also associated with lower rates of labor induction and episiotomy, higher vaginal birth rates, and increased maternal satisfaction (17,21). Leeman and Leeman determined that family medicine physicians and certified nurse-midwives were predominantly involved in Zuni and Navajo births. This involvement may be associated with less use of cesarean delivery for labor dystocia and high rates of vaginal delivery after cesarean, which could partially account for the low rate of cesarean delivery despite the high incidence of diabetes and preeclampsia in this population. Furthermore, cultural attitudes toward childbirth and the design of the perinatal system may have also contributed to the low 7.3% cesarean delivery rate (16).

### Study quality

The randomized controlled trial (19) met 9 of 13 Joanna Briggs Institute criteria (moderate quality); the moderate rating was primarily due to limited reporting on blinding procedures and allocation concealment (Table 3). Among the cohort studies, Newcastle–Ottawa Scale scores ranged from 7 to 9 (good quality) (Table 4). These studies generally demonstrated strong cohort selection methods, appropriate ascertainment of exposure and outcomes, and adequate follow-up. The study by Stone et al (17) received a slightly lower score than the others because of limited adjustment for confounding variables. Overall, the included literature demonstrated moderate to high methodologic rigor.

**Table 3:** Joanna Briggs Institute Quality Assessment of Single Randomized Controlled Trial Included in Systematic Review of Studies Describing Interventions for Reducing Rates of Cesarean Delivery in Rural US Communities^a^

Question no.	Question	Judgment
1	Was true randomization used?	Yes
2	Was allocation concealed?	Some concerns/cannot determine
3	Were groups similar at baseline?	Yes
4	Were participants blinded?	No
5	Were those delivering treatment blinded?	No
6	Were outcome assessors blinded?	No
7	Were groups treated identically other than the intervention?	Yes
8	Was follow-up complete?	Yes
9	Were participants analyzed in the groups to which they were randomized?	Yes
10	Were outcomes measured in the same way for groups?	Yes
11	Were outcomes measured reliably?	Yes
12	Was appropriate statistical analysis used?	Yes
13	Was the trial design appropriate?	Yes

a Feinberg et al (19), 2015; 9 of 13 criteria were met, indicating moderate quality.

**Table 4:** Newcastle–Ottawa Scale Quality Assessment of Studies Included in Systematic Review Describing Interventions for Reducing Rates of Cesarean Delivery in Rural US Communities^a^

Study	No. of items met				Quality rating
	Selection	Comparability	Outcome	Total	
Bush et al (20), 2017	3	2	3	8	Good
Carlough et al (18), 2021	3	2	3	8	Good
Hueston and Rudy (15), 1993	4	2	3	9	Good
Prasad et al (6), 2018	4	2	3	9	Good
VanGompel et al (14), 2024	4	2	3	9	Good
Leeman and Leeman (16), 2003	4	2	3	9	Good
Jolles et al (21), 2020	4	2	3	9	Good
Stone et al (17), 1996	3	1	3	7	Good

a Criteria include the following. For selection: 1) representativeness of the exposed cohort, 2) selection of the nonexposed cohort, 3) ascertainment of exposure, 4) outcome not present at start of study. For comparability: 1) study controls for the most important confounder, 2) study controls for additional confounders. For outcome: 1) assessment of outcome, 2) adequacy of follow-up duration, adequacy of follow-up completeness.

## Discussion

This systematic review identified promising approaches to reduce rates of cesarean delivery in rural US communities. Patient-level strategies alone had a variable effect on cesarean delivery rates. The mobile health app was associated with increased prenatal engagement, but did not meaningfully reduce cesarean delivery rates, and the study was compromised by low participation rates (20). The couples-based psychosocial intervention demonstrated a large absolute difference (19%) in cesarean delivery rates compared with usual care, but it was tested on a small sample, and the study did not discuss program sustainability of the 9-session counseling model (19).

By contrast, system-level interventions that examined staffing models, midwifery-led care, and maternity systems were consistently associated with lower cesarean delivery rates compared with conventional models. Prasad et al reported a 3.9% lower rate of NTSV cesarean deliveries and a 4.0% lower rate of non–medically indicated cesareans in hospitals directly employing family medicine physicians (rather than contracting with them) (6), while VanGompel et al reported a 34.3% lower adjusted risk of NTSV cesarean delivery in hospitals staffed only by family medicine physicians compared with hospitals staffed by both family medicine physicians and obstetricians (14). Additional studies demonstrated meaningful absolute differences, including lower cesarean delivery rates among patients managed by certified nurse–midwives (8%) versus family medicine physicians (14%) (15), and substantial reductions observed after implementation of collaborative care models integrating midwives, nurses, and obstetricians (26.2% in 1966 to 11.2% in 1994) (17). These findings suggest that, while patient education or psychosocial programs can meaningfully support individual birthing experiences, supportive staffing structures, organizational norms, and clinical decision-making cultures may play a bigger role in achieving sustained reductions in cesarean delivery rates.

The highlighted system-level models were also associated with other positive outcomes, including lower episiotomy rates and higher success in vaginal birth after cesarean delivery. This finding suggests that the integration of family medicine physicians and certified nurse–midwives, whose training and scope of practice often emphasize continuity of care and support for physiologic labor, may be associated with less intervention-intensive labor management and lower rates of cesarean delivery. These findings should not be interpreted as differences in provider quality among family medicine physicians, certified nurse–midwives, and obstetricians, but rather as the influence of care models, staffing structures, and scope-of-practice alignment on delivery outcomes.

While system-level models emphasizing midwifery integration and family medicine physician–led care were consistently associated with lower cesarean delivery rates, these findings should be interpreted within the context of rural workforce limitations. Many rural communities lack sufficient maternity care infrastructure, including shortages of physicians, certified nurse–midwives, and anesthesia services. As a result, the feasibility of implementing or scaling these models may vary substantially depending on local workforce capacity, referral networks, and access to higher-level care.

A consideration when interpreting these findings is the potential for structural and referral-related confounding. Rural hospitals with lower cesarean delivery rates may differ from higher-rate hospitals in ways not fully captured in the included studies, including patient risk profiles, referral patterns, and regional practice norms. For example, facilities with limited surgical capacity or those that transfer higher-risk pregnancies to tertiary centers may appear to have lower cesarean rates independent of care model effects. Similarly, differences in patient case mix, such as the proportion of high-risk pregnancies, may influence observed outcomes. These factors limit the ability to attribute differences in cesarean delivery rates solely to staffing models or system design.

Reductions in cesarean delivery rates do not necessarily reflect improved quality of care. In rural settings, limited access to surgical services may delay or restrict necessary interventions for high-risk pregnancies, potentially resulting in adverse outcomes. This is particularly important to consider in the absence of detailed information on patient cases and clinical indications.

Although no included studies directly evaluated provider-level or broader policy-level interventions, these approaches provide context for interpreting the findings of this review. National guidelines from the American College of Obstetricians and Gynecologists (ACOG) and quality-improvement initiatives such as the Alliance for Innovation on Maternal Health Safe Reduction of Primary Cesarean Birth support standardized labor management protocols, evidence-based definitions of labor progress, audit-and-feedback systems, team-based care, and maintenance of clinical skills, all of which have been associated with reduced cesarean delivery rates (22,23). For example, adherence to updated labor management guidelines, including defining active labor at 6-cm dilation and requiring stricter criteria before diagnosing labor dystocia, have been associated with lower cesarean delivery rates (22). At the systems-level, payer and Medicaid reforms, public reporting requirements, and equalized reimbursement for vaginal and cesarean births have also been proposed to address structural drivers of cesarean delivery (22). Although emerging evidence from nonrural settings suggests these strategies may be promising, no studies in this review evaluated their implementation or effectiveness in rural communities. Additional research is needed to understand how rural conditions may influence the feasibility and impact of these provider- and systems-level approaches.

Promoting genuine collaboration and team-based models among obstetricians, family medicine physicians, and certified nurse–midwives is a strategy associated with reduced cesarean delivery rates (17). Obstetricians are essential for managing surgical and high-risk obstetric conditions, whereas family medicine physicians and certified nurse–midwives often emphasize support of physiologic labor for low-risk pregnancies. Integrating these complementary skill sets through clinical guidelines and practice incentives may help reduce reliance on operative delivery, preserve access to surgical expertise when clinically indicated, and increase availability of continuous labor support and nonoperative management options. Given growing evidence that cesarean delivery is associated with increased risk of long-term maternal and infant morbidity, collaborative care models may be associated with more deliberate and evidence-based use of operative intervention while preserving patient safety.

An important contextual example comes from the Leeman and Leeman study in a Zuni community, which illustrates how multilevel alignment across health system design, health care provider decision-making, and culturally grounded childbirth practices can produce exceptionally low cesarean delivery rates (16). In this community, because cesarean delivery required transfer to a distant hospital, 2 physicians were required to reach consensus that a surgical emergency was present. This built-in consultation process may have functioned as a structural safeguard against nonemergent operative delivery and may have reduced provider-level variation in surgical decision-making. Additionally, the authors noted that Zuni women preferred to avoid obstetric intervention and that female relatives commonly provided continuous support during labor and delivery, which may also have contributed to birth outcomes (16). These labor companions may function in roles similar to doulas, providing continuous emotional and physical support during labor. Consistent with this, a 1999 meta-analysis found that continuous labor support was associated with shorter labors and decreased use of analgesia, oxytocin, forceps, and cesarean delivery (24). These findings parallel those from the psychosocial intervention described by Feinberg et al, in which enhanced emotional support for expectant couples was associated with a meaningful reduction in cesarean deliveries (19). Together, these findings suggest that reductions in unnecessary cesarean delivery may be most achievable when systems-level safeguards are paired with patient-centered, culturally responsive forms of labor support.

### Recommendations

Based on this review’s findings, as well as recommendations from national best-practice reports (25) and studies from urban settings (26), the most promising strategy for reducing rates of unnecessary cesarean delivery in rural communities is to integrate culturally responsive, patient-centered support models with systems-level redesign. Actionable strategies can be organized into immediate, intermediate, and long-term priorities, as well as by level of resource availability.

Immediate (low-resource, high-feasibility) strategies include implementing standardized labor management protocols aligned with ACOG guidelines, introducing audit-and-feedback systems to track cesarean delivery rates at the health care provider and facility level, and expanding access to continuous labor support. Supporting culturally congruent labor companionship (eg, doulas and trusted family members) may be a feasible patient-centered strategy to improve labor experiences and reduce intervention intensity in rural communities, particularly in communities where such support structures are already embedded within local cultural practices.

Intermediate (moderate-resource, system adaptation) priorities include developing collaborative care models integrating family medicine physicians, certified nurse–midwives, and obstetricians, strengthening referral networks and transfer protocols, and piloting psychosocial support interventions that enhance patient engagement and reduce stress-related risk factors. Long-term (high resource and structural) strategies require investment in workforce development to expand the rural maternity care workforce, support for midwifery-led birth centers and integrated maternity care systems, and alignment of reimbursement and policy incentives to support physiologic birth. Although expanding midwifery services appears particularly promising, it is often undervalued due to regulatory, financial, and structural power imbalances within medical systems (21,27).

Change also depends on the capacity of rural health centers. In lower-capacity settings, protocol standardization, telehealth support, and transfer systems should be prioritized. Moderate-capacity settings should aim to implement collaborative staffing models and targeted quality improvement initiatives. Higher-capacity rural systems should expand midwifery-led models and integrated team-based care. These tiered recommendations emphasize that reducing unnecessary cesarean delivery in rural settings requires alignment across clinical practice, workforce capacity, and health system design.

### Limitations

This review has several limitations. First, many of the interventions included in this review were not inherently rural in design and could feasibly be applied in urban settings. Thus, it is challenging to determine which aspects of these interventions were particularly effective in rural contexts or whether they addressed rural-specific barriers such as workforce shortages, geographic isolation, or cost constraints. For example, while midwifery-led models were among the most effective models, the studies provided limited details about how these models were implemented in low-resource rural areas, where costs and staffing limitations may hinder scalability.

Second, this review excluded gray literature, such as program evaluations and community-based program documentation. This may be an important limitation, particularly in rural settings, where innovative maternity care strategies are often developed and implemented by local health departments, tribal or Indigenous health systems, community organizations, and small rural hospitals without being formally evaluated in peer-reviewed journals. As a result, this review may underrepresent promising real-world practices and overrepresent interventions originating from academic or research-intensive settings. Future reviews should consider including gray literature to better capture community-driven and practice-based innovations relevant to rural maternity care.

Third, included studies used various criteria (eg, county size, distance to obstetric services, number of annual deliveries, or federal rural–urban classification codes) to designate a community as rural. Thus, the studies may represent heterogeneous populations with differing levels of access to care, geographic isolation, and resource availability, creating a challenge for comparing results. Future research would benefit from applying standardized rural classification frameworks to improve consistency and interpretability across studies.

### Conclusion

Evidence from this systematic review suggests that system-level factors, including staffing models, midwifery integration, involvement of family medicine physicians, culturally grounded childbirth practices, and continuous labor support, rather than patient-level interventions alone, are consistently associated with lower rates of cesarean delivery in rural US communities. Efforts to reduce unnecessary cesarean delivery in rural settings should prioritize context-specific implementation of collaborative, culturally responsive care models aligned with local workforce capacity. Further research is needed to evaluate how these approaches can be effectively adapted and sustained across diverse rural settings.

## Appendix Full Database Search Strategies for Systematic Review of Studies Describing Interventions for Reducing Rates of Cesarean Delivery in Rural US Communities

**PubMed**

(Intervention OR strategies OR programs OR approaches OR practices OR initiatives OR measures) AND (“Cesarean Section”[Mesh] OR “Cesarean Section” OR C-section OR “Caesarean Section” OR “Cesarean delivery” OR “Surgical birth”) AND (“Rural Population”[Mesh] OR “rural communities” OR “rural areas” OR “rural populations” OR remote OR frontier OR non-metropolitan OR “small towns” OR “underserved areas” OR “health professional shortage areas” OR HPSA OR “outlying areas” OR “low-resource areas” OR “sparsely populated”) AND (“United States”[Mesh] OR USA OR US OR U.S. OR national OR America OR American).

**CINAHL**

(Intervention OR strategies OR programs OR approaches OR practices OR initiatives OR measures) AND ((MH “Cesarean Section+”) OR “Cesarean Section” OR C-section OR “Caesarean Section” OR “Cesarean delivery” OR “Surgical birth”) AND ((MH “Rural Population+”) OR “rural communities” OR “rural areas” OR “rural populations” OR remote OR frontier OR non-metropolitan OR “small towns” OR “underserved areas” OR “health professional shortage areas” OR HPSA OR “outlying areas” OR “low-resource areas” OR “sparsely populated”) AND ((MH “United States+”) OR USA OR US OR U.S. OR national OR America OR American).

**Cochrane Library**

(Intervention OR strategies OR programs OR approaches OR practices OR initiatives OR measures ) AND ([mh “Cesarean Section”] OR “Cesarean Section” OR C-section OR “Caesarean Section” OR “Cesarean delivery” OR “Surgical birth” ) AND ([mh “Rural Population”] OR “rural communities” OR “rural areas” OR “rural populations” OR remote OR frontier OR non-metropolitan OR “small towns” OR “underserved areas” OR “health professional shortage areas” OR HPSA OR “outlying areas” OR “low-resource areas” OR “sparsely populated” ) AND ([mh “United States”] OR USA OR US OR U.S. OR national OR America OR American).

**Web of Science Core Collection**

(Intervention OR strategies OR programs OR approaches OR practices OR initiatives OR measures ) AND (“Cesarean Section” OR “Cesarean Section” OR C-section OR “Caesarean Section” OR “Cesarean delivery” OR “Surgical birth” ) AND (“Rural Population” OR “rural communities” OR “rural areas” OR “rural populations” OR remote OR frontier OR non-metropolitan OR “small towns” OR “underserved areas” OR “health professional shortage areas” OR HPSA OR “outlying areas” OR “low-resource areas” OR “sparsely populated” ) AND (“United States” OR USA OR US OR U.S. OR national OR America OR American).
